# Dracula's Menagerie Reloaded: Assessing the Relative Roles of Habitat and Interspecific Interactions in an Intact Mammalian Assemblage Using Structural Equation Modeling

**DOI:** 10.1002/ece3.71381

**Published:** 2025-04-25

**Authors:** Marissa A. Dyck, Ruben Iosif, Barbara Promberger–Fürpass, Viorel D. Popescu

**Affiliations:** ^1^ School of Environmental Studies University of Victoria Victoria British Columbia Canada; ^2^ Foundation Conservation Carpathia Brașov Romania; ^3^ Center for Environmental Research University of Bucharest Bucharest Romania; ^4^ Department of Ecology, Evolution and Environmental Biology Columbia University New York New York USA

## Abstract

Interspecific interactions play a central role in structuring animal communities and food webs. In particular, carnivores are important topdown regulators in ecological communities and the loss of carnivore species can have devastating ecosystem effects. Similarly, carnivore reintroductions are successful if the prey base is sufficient to support population growth, making the case for the importance of bottom‐up regulation processes. As such, rewilding efforts targeted at restoring food webs and natural community regulation processes (trophic rewilding) have become increasingly popular. However, investigations of regulation processes in terrestrial vertebrate communities often take place in heavily altered systems, potentially biasing inference on the presence or importance of top‐down versus bottom‐up regulation processes. Here, we use a stable mammalian assemblage in the Romanian Carpathians to evaluate the relative importance of top‐down and bottom‐up processes and provide a benchmark for understanding the effects and the success of rewilding initiatives. To do so, we used camera trap data from two consecutive years in the Southern Romanian Carpathians and developed hypothesisbased interaction pathways for top‐down and bottom‐up regulation in a piecewise structural equation modeling (SEM) framework. Results from SEMs indicate that while both top‐down (wolf and Eurasian lynx‐driven) and bottom‐up processes (driven by roe deer, red deer, wild boar and hare abundance) play important roles in shaping community structure, landscape characteristics (i.e., terrain ruggedness, road density, elevation, and forest cover) have a greater effect on both predators and prey. The results of this research have implications for rewilding efforts in Europe and globally. This study highlights the importance of preserving natural habitats, underscoring that effective species conservation and coexistence must go hand in hand with conserving natural spaces.

## Graphical abstract

We used an intact mammalian assemblage in the Romanian Carpathians to evaluate the relative importance of top‐down and bottom‐up processes and provide a benchmark for understanding the effects and the success of rewilding initiatives. We used camera trap data from two consecutive years in the Southern Romanian Carpathians and developed hypothesis‐based interaction pathways for top‐down and bottom‐up regulation in a piecewise structural equation modeling (SEM) framework. Results from SEMs indicate that while both top‐down (wolf and Eurasian lynx‐driven) and bottom‐up processes (driven by roe deer, red deer, wild boar and hare abundance) play important roles in shaping community structure, landscape characteristics (e.g., forest cover, ruggedness) have a greater effect for both predators and prey.

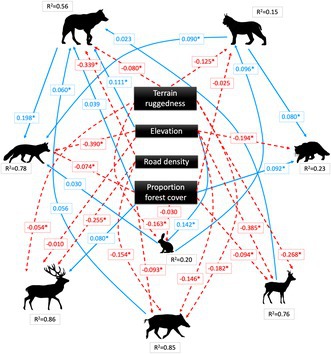

## 1 Introduction

Interspecific interactions play a central role in structuring plant and animal communities and food webs (Connell 1983, May 1983). The ecological processes resulting from interspecific interactions and wildlife‐habitat relationships can be classified as top‐down and bottom‐up, and there is variation in published literature as to the relative importance of these processes in shaping ecosystems. In the context of predators, bottom‐up processes are driven by the abundance and distribution of prey species and their food and habitat resources, which have been shown to impact and regulate the behavior, abundance, and distribution of predators (Blüthgen et al. 2004; Burkepile et al. 2013; Cano‐Martínez et al. 2024). Top‐down processes are primarily driven by top or apex predators, where predation pressure alters the behavior, spatial distribution, and composition of prey species, smaller predators (e.g., mesopredators), and communities through direct and indirect effects (e.g., landscape of fear) (Sinclair 1985; Matassa and Trussell 2011; Levi and Wilmers 2012; Kohl et al. 2018). In some cases, top‐down processes can cascade through multiple trophic levels and alter the abundance and distributions of primary producers and ecosystem dynamics (Bowman et al. 2001; Shurin et al. 2002; Letnic et al. 2009; Madin et al. 2011; Ripple and Beschta 2012; Strickland et al. 2013; Terborgh and Estes 2013).

Apex predators are often considered to exert strong top‐down regulation on ecosystems, and research has shown that the loss of these species can lead to substantial increases in herbivores (Berger et al. 2001; Hebblewhite et al. 2005) and mesopredators (i.e., mesopredator release) (Crooks and Soulé 1999; Elmhagen and Rushton 2007; Ritchie and Johnson 2009; Levi and Wilmers 2012). Increased abundance of herbivores and mesopredators can result in large‐scale negative impacts such as depletion of biomass resources and suppression of smaller prey species (Crooks and Soulé 1999; Terborgh et al. 1999; Berger et al. 2001; Hebblewhite et al. 2005). Thus, trophic rewilding—the recovery of apex predators to restore ecosystem structure and function—has gained popularity as a conservation tool (Pace et al. 1999; Boitani and Linnell 2015; Svenning et al. 2016; Cromsigt et al. 2018; Schweiger et al. 2019).

However, research has also shown that bottom‐up effects can play a large role in shaping ecosystems and, in some cases, can be more influential than top‐down effects (Paradise and Dunson 1997; Frederiksen et al. 2006; Keeler et al. 2006; Elmhagen and Rushton 2007; Scherber et al. 2010; Allen et al. 2012; Stoessel et al. 2019). Furthermore, previous research suggests that the nature of predator–prey interactions is likely to be highly context‐dependent (Kuijper et al. 2016; Gigliotti et al. 2020). The relative strength of bottom‐up and top‐down processes in mammal communities has often been studied in systems that underwent reintroductions, recolonizations, or expansion, often following conservation measures (Ripple and Beschta 2006, 2012; Ripple et al. 2014). However, there is less research on the importance of top‐down processes in systems that have not experienced extinctions/extirpations or reintroductions, and fewer studies have assessed trophic relations and processes that structure animal communities in human‐dominated landscapes (e.g., Dorresteijn et al. 2015; Kuijper et al. 2016). These systems, in which the animal community is actively managed (e.g., via hunting or conservation measures), but remained stable over centuries, likely represent benchmarks for understanding long‐term community dynamics. As such, understanding processes that structure animal communities in these systems can provide insights into the potential effects of rewilding and self‐recolonization on existing and often depauperate recipient animal communities in adjacent systems in which extirpations occurred historically.

Europe represents a great case study for this research, as large carnivores inhabit or are recolonizing ecosystems that are heavily modified by humans (Chapron et al. 2014). Historically, apex predator abundance and distribution declined throughout Europe, with many species suffering severe range contractions or—as was the case with many populations of large carnivores in Western Europe—complete extirpation (Trouwborst 2010; Ripple et al. 2014; Wolf and Ripple 2017; Boitani 2000; Breitenmoser 2000; Landa et al. 2000; Swenson 2000). In recent decades, apex predators have returned to human‐dominated European landscapes (Chapron et al. 2014) either naturally (e.g., wolf) or through translocations and population augmentation (lynx and brown bear), yet there is limited data on the effects of recolonization on the recipient mammalian communities, often dominated by mesocarnivores and overabundant ungulate populations (Valente et al. 2020). For example, in many parts of Europe apex carnivores have been extirpated for centuries (Kuijper et al. 2016) and prey may therefore be naïve to the risks of predators (Berger et al. 2001). Similarly, the effects of large carnivores, particularly wolves, on livestock in newly recolonized areas of Europe (e.g., France, Germany, Italy) are leading to increased human‐wildlife conflict (Davoli et al. 2022). As such, an improved understanding of the relation between carnivores and ungulates inferred from community‐level analyses in intact systems can provide a platform for potentially alleviating human‐wildlife conflict.

The natural landscapes of Southern Carpathians (Romania) offer an ideal location for studying top‐down and bottom‐up processes in a community that harbors one of the most intact assemblages of mammalian carnivores and herbivores in temperate Europe. Importantly, only one mammalian species in this system was extirpated, the European bison or wisent ( *Bos bonasus* ), which disappeared in the 1800's. European Bison played a significant role in this forest ecosystem with herbivory and predator–prey relations, and efforts to restore ecosystem function via bison translocations are currently underway in the Romanian Carpathians (Tănăsescu 2019; Dănilă et al. 2022), including in the study region (R. Iosif, pers. comm) although translocations did not overlap the study period. All other temperate mammalian species have large, stable and genetically diverse populations (Popescu et al. 2017; Sin et al. 2017; Iosif et al. 2021, 2022). While several mammalian species are managed as game species (e.g., ungulates, some mesocarnivores), apex predators are protected under European Union (EU) Habitats Directive, and hunting is not permitted (this does not include brown bear, *Ursus arctos*, for which hunting has resumed in 2024 after an eight‐year ban; Pop et al. 2025). Additionally, despite generally low human population density, there is substantial human influence on the Carpathian landscape (i.e., logging, recreation, hunting, livestock grazing), which affects spatial and temporal patterns of both carnivore and ungulate distributions (Salvatori et al. 2002; Pop et al. 2018, 2023; Dyck et al. 2022; Iosif et al. 2022). Thus, studying predator–prey interactions and interspecific competition in the Romanian Carpathians system provides a unique opportunity to improve our understanding the relative importance of top‐down and bottom‐up processes in long‐term structuring of a stable and diverse European temperate mammalian community. As such, lessons learned from the Carpathian system have the potential to yield insights into the effects of carnivore recolonization on the depauperate mammalian communities in other parts of Europe and serve as a benchmark for measuring the effect of carnivore recolonization on local communities across Europe.

We used two seasons of motion‐triggered camera trap data with piecewise structural equation modeling (SEM) to assess the relative importance of top‐down versus bottom‐up processes in shaping the apex predator, mesopredator, and prey community in the Romanian Carpathians. SEMs are used to test hypothesized causal relations between multiple predictor and response variables (Pearl 2000; Karimi and Meyer 2014), and are particularly useful for quantifying indirect and cascading effects as variables can serve as both predictors and responses in the model framework (Lefcheck 2016). Using this framework, we constructed networks of pairwise relationships between predators, prey, and landscape characteristics using known relationships derived from the European and North American scientific literature (see *Hypothesized SEM Pathways*), and from previous research in this system (Sin et al. 2019; Dyck et al. 2022; Iosif et al. 2022) as separate Directed Acyclic Graphs representing pure top‐down and bottom‐up processes, as well as a combined framework (i.e., containing both top‐down and bottom‐up) derived from the two other models. Specifically, our objectives were: (1) to identify the simple network explaining both top‐down and bottom‐up regulation in the Carpathian large and meso‐mammalian community, (2) to determine key species or landscape characteristics driving top‐down and bottom‐up models, as well as non‐interacting species, and (3) to identify the model that best explains mammalian interactions in the Romanian Carpathians. This study has the potential to provide a baseline understanding of how intact mammalian communities function, identify determinants of species coexistence or exclusion, and contribute to mammalian conservation efforts in Europe.

## 2 Materials and Methods

### 2.1 Study Area

**FIGURE 1:**
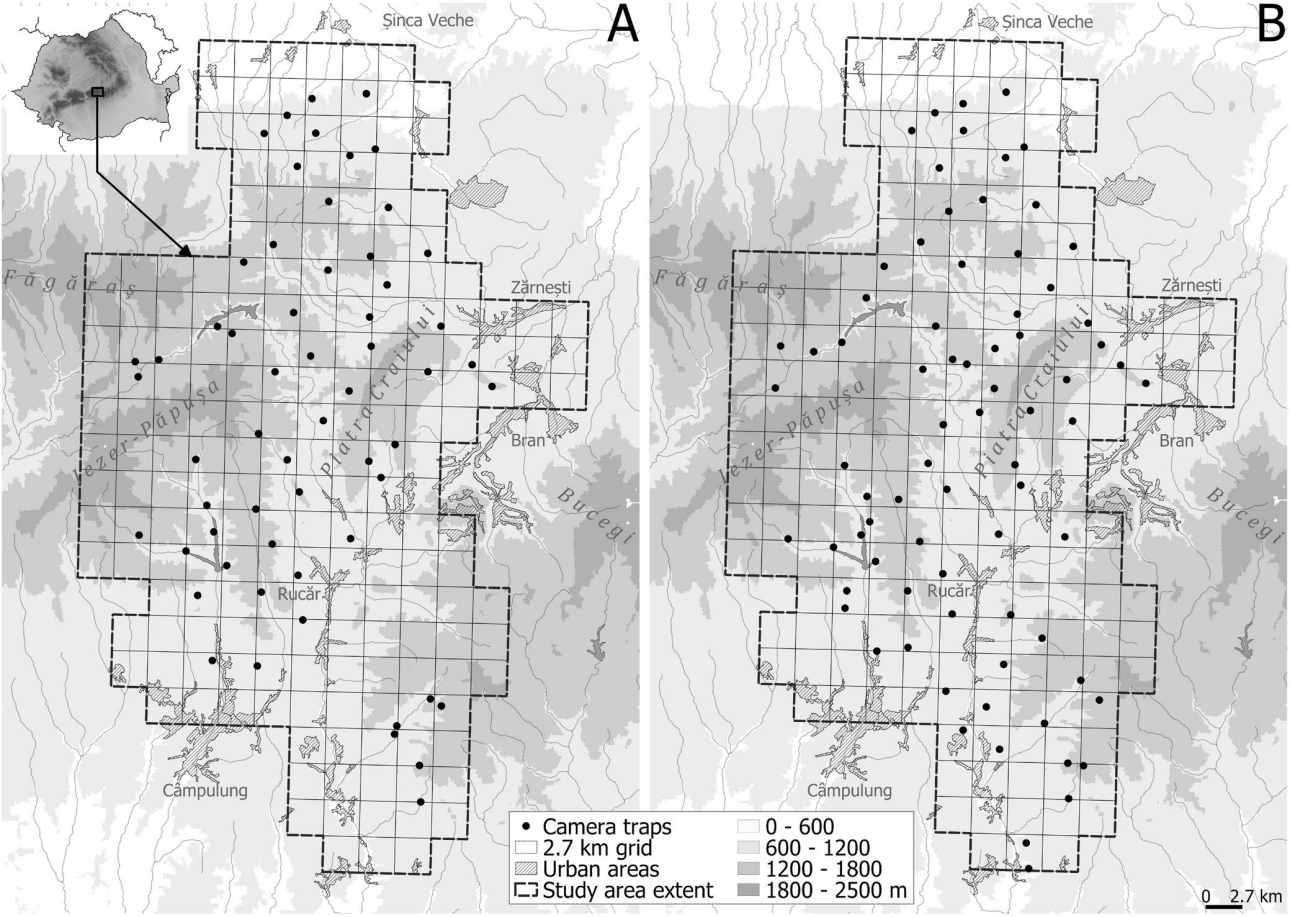
Study area for winter (A) and autumn (B) sessions and the locations of 64 (winter) and 76 (autumn) camera trap stations in Romanian Carpathians used for camera trap surveys of Eurasian lynx ( *Lynx lynx* ), European wildcat ( *Felis silvestris* ), and gray wolf ( *Canis lupus* ). Camera sessions lasted from December 17th, 2018, to March 31st, 2019 (winter) and October 9th, 2019, to January 15th, 2020 (autumn). Data from both seasons were included in a single piecewise structural equation model (SEM) to assess the relative importance of top‐down and bottom‐up processes in shaping community dynamics.

The study area is situated in the southern Carpathians, Romania, covering 1200 km^2^ in the eastern part of the Făgăraș Mountains, Piatra Craiului, and parts of the Leaota Mountains (Figure 1). The study area's habitat is dominated by forest stands (62%), with urban–rural landscapes and agriculture mixed with natural vegetation (22%), and alpine grasslands and subalpine shrubs (16%) (Dyck et al. 2022; Iosif et al. 2022). Elevation ranges from 600 to 2400 m and the area is bisected by a high traffic national road with a network of unpaved and temporary logging roads (Dyck et al. 2022; Iosif et al. 2022). Forest composition is dominated by European beech ( *Fagus sylvatica* ) at lower elevations (< 1200 m), which transitions into mixed forests of beech‐fir‐spruce ( *F. sylvatica* , *Albies alba* and *Picea abies* ) and coniferous spruce‐fir forests at high elevations (1400–1800 m). Natural grasslands are prevalent in this landscape at elevations above 1000 m, with alpine and subalpine grassland and shrubland above 1800 m. The mean annual temperature at the highest elevations is 0°C and increases to 7°C–8°C at lower elevations. The precipitation ranges between 900 mm at the lowest elevation and 1300 mm at the highest. Above the tree line, consistent snowpack occurs between December and April, while below 1600 m, the snowpack can fluctuate between 0 and 50 cm within short periods.

The Romanian Carpathians are recognized as one of the strongholds for large carnivores and ungulates in Europe and as a critical a corridor for dispersal between central and southern European wildlife populations. The area harbors a diverse mammal assemblage including some of the highest densities of apex carnivores in Europe: brown bear ( *Ursus arctos* ; density estimate: 17.76 bears/100 km^2^ [95% CI: 15.40–25.74]) (Iosif et al. 2021), Eurasian lynx ( *Lynx lynx* , hereafter lynx; density estimate: 1.6 ± 0.39 SE and 1.7 ± 0.38 SE lynx/100 km^2^, for winter and autumn respectively) (Iosif et al. 2022), gray wolf ( *Canis lupus* , hereafter wolf; density estimates: 1.00–2.80 wolves/100 km^2^ and 2.34 wolves/100 km^2^ [Bayesian Credible Interval: 1.68–3.03]) (Sin et al. 2017). Hunting of ungulates, such as roe deer ( *Capreolus capreolus* ), red deer ( *Cervus elaphus* ), chamois ( *Rupicapra rupicapra* ), and wild boar ( *Sus scrofa* ) occurs in the study area; however, hunting of carnivores has not occurred during the study period. Logging remains an important economic activity and occurs at low density year‐round throughout the study area. Agriculture is another source of human impact, and livestock grazing is prevalent in the alpine areas, whereas lowlands are characterized by small‐scale subsistence farming and occasional tourism development. The study was part of research conducted by Foundation Conservation Carpathia (FCC) to evaluate the occurrence and abundance of large carnivores (primarily lynx) and their prey in the Southern Romanian Carpathians (Dyck et al. 2022; Iosif et al. 2022).

### 2.2 Data Collection

Study design and camera trap implementation follows methods outlined in Dyck et al. (2022). Briefly, we divided the study area into 2.7 km x 2.7 km grid cells (Figure 1) and removed cells deemed unsuitable for focal species inhabitance (e.g., more than ⅔ of their area exceeding 1800 m elevation and more than ½ of their area covered by urban landscape features). Cameras were then deployed randomly in every other cell; if a cell was inaccessible, an adjacent cell was used. We conducted two seasons of monitoring: winter (64 cameras) and autumn (76 cameras), which were combined for modeling. The winter season lasted from 17 December 2018 to 31 March 2019, and the autumn season from 9 October 2019 to 15 January 2020. Cameras were installed 1–2 weeks prior to the start of data collection to allow for an acclimation period and account for the additional anthropogenic disturbance from the setup process. We deployed two cameras (height 40–60 cm) opposite each other at each location along animal paths at various elevations/habitats. Two camera models were used at each location: a CuddeBack C1 Model 1279 with white flash for high‐quality color pictures in night conditions and a Bushnell Trophy infrared camera. Cameras were checked every 2 weeks to replace batteries and SD cards. Data collection periods and camera deployment protocol and design were primarily targeted at estimating the density of lynx in the Romanian Carpathians (Iosif et al. 2022), following recommendations of Zimmermann et al. (2013). As such, this study period largely overlapped with the hibernation season for brown bears, a common large carnivore species in this region, and thus did not include them in the analysis.

We calculated the occurrence of eight species for each season: apex carnivores: lynx and wolf; mesocarnivores: red fox ( *Vulpes vulpes* , hereafter fox) and European wildcat ( *Felis silvestris* , hereafter wildcat); and herbivores/prey: European hare ( *Lepus europaeus* , hereafter hare), roe deer, red deer, and wild boar. Species occurrences were calculated by summing all records for each species per camera location (Dorresteijn et al. 2015). At each camera location, we recorded *elevation* in meters and used Geographic Information Systems (GIS) to extract additional landscape data, including the proportion of forested area (*forest*), the density of local roads (*road density*; km/km^2^), and the terrain ruggedness index (*TRI*; Riley et al. 1999) which were calculated within a 500‐m buffer around each camera trap location to ensure comparability with previous studies in the system (Dyck et al. 2022). Full descriptions of landscape variables can be found in Table 1. All landscape variables were scaled and centered for data analysis, and pairwise tests of Pearson's correlation were conducted to ensure independence of variables; all correlations were low (*r* < 0.25).

**TABLE 1:** Names and descriptions of environmental variables used in piecewise structural equation models (SEM) predicting causal pathways of species occurrence data from camera traps for Eurasian lynx ( *Lynx lynx* ), gray wolf ( *Canis lupus* ), European wildcat ( *Felis silvestris* ), red fox ( *Vulpes vulpes* ), European hare ( *Lepus europaeus* ), red deer ( *Cervus elaphus* ), roe deer ( *Capreolus capreolus* ), and wild boar ( *Sus scrofa* ) in the Romanian Carpathians.

Name	Description	Type	Summary data
Local road density	Density of roads (km/km^2^) at the grid cell level	Numeric variable ranging from 0.21 to 0.34 km/km^2^	Mean = 0.27 km/km^2^
Terrain Ruggedness Index (TRI)	TRI was calculated in R via package ‘*spatialEco*’ using a digital elevation model with resolution 80 × 80 m and two moving window sizes: five cells (covering an area of 0.16km^2^)	Numeric variable ranging from 84 to 494 with recommended classification ranges 81–116 = nearly level surface 117–161 = slightly rugged surface 162–239 = intermediately rugged surface 240–497 = moderately rugged surface	Mean = 220.27
Proportion forest	Proportion of forest at the grid cell level (extracted from Corine Land Cover 2016 dataset [100 × 100 m resolution] using GIS)	Numeric variable ranging from 0.1 to 1.0	Mean = 0.77
Elevation	Elevation of the camera location recorded in meters using GPS by field personnel	Numeric variable ranging from 663 to 1617 m	Mean = 1166 m

### 2.3 Model Development

We used piecewise Structured Equation Models to assess the relative importance of top‐down versus bottom‐up influences in shaping the mammalian community in the Romanian Carpathians. Piecewise SEM differs from traditional SEM in that the path diagrams are represented by a set of linear (structured) equations which are evaluated individually rather than simultaneously as with traditional SEM (Lefcheck 2016). This transition from global estimation to local estimation allows for data to be fit with a wide range of distributions and smaller datasets (Shipley 2000, 2009; Lefcheck 2016).

Model development and analysis were conducted in program R version 4.2.1 (R Core Team 2022) using the ‘peicewiseSEM’ package (Lefcheck 2016). We modeled species occurrences as endogenous variables (response variables influenced by other covariates in the model) and environmental covariates as exogenous variables (independent variables not influenced by other covariates in the model). We utilized a stepwise approach to develop three models, a top‐down model, a bottom‐up model, and a model which included both top‐down and bottom‐up pathways. All models included multiple generalized linear models (GLMs) fit to a Poisson distribution as structured equations for occurrence of each species. We first developed separate a priori top‐down and bottom‐up models that represented hypothesized causal pathways among species and between species and environmental variables.

### 2.4 Hypothesized Pathways

#### 2.4.1 Top‐Down Processes

**FIGURE 2:**
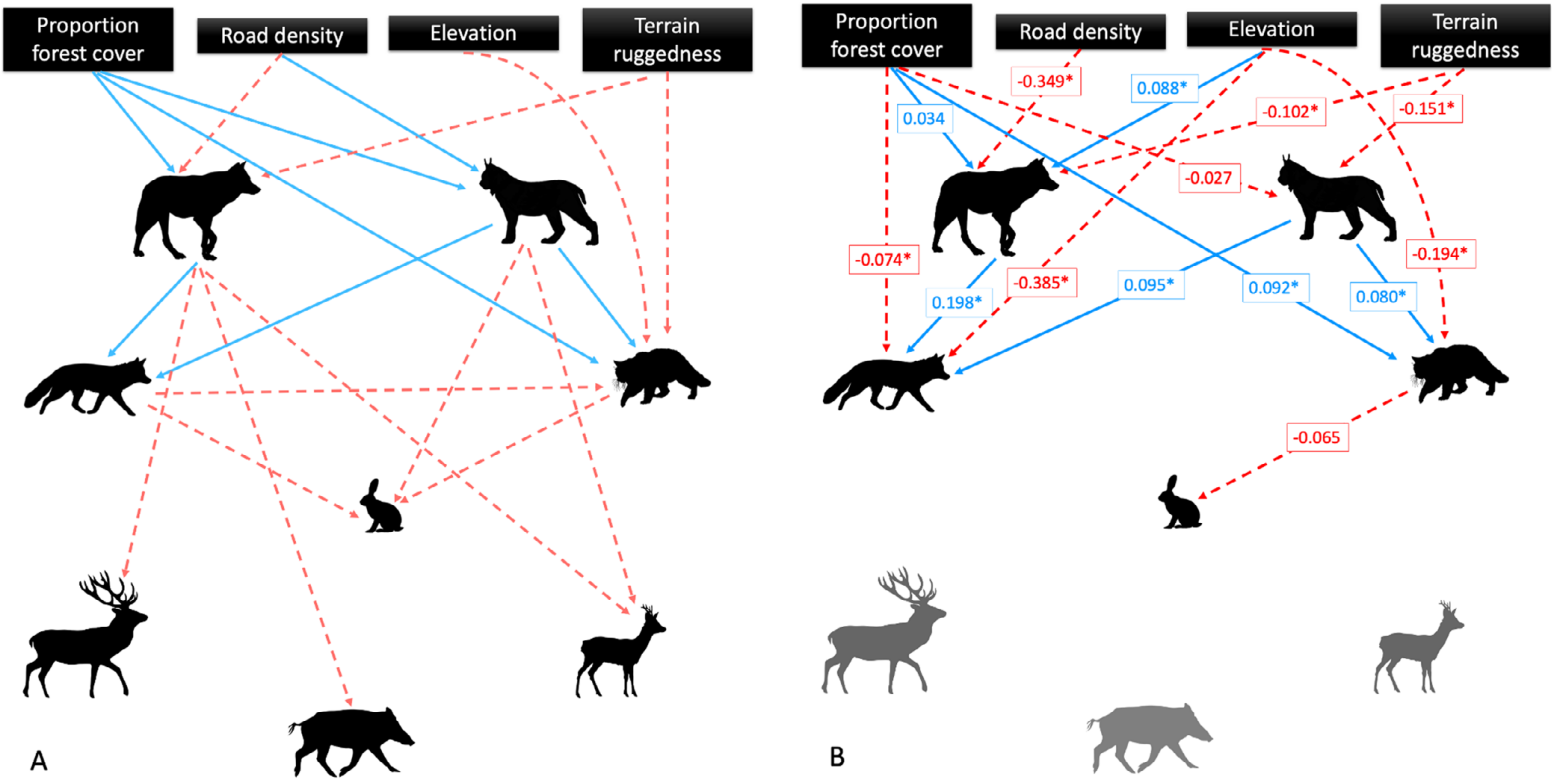
Hypothesized (A) and optimized (B) piecewise structural equation models (SEM) for top‐down species interaction and landscape characteristics in the Romanian Carpathians. Red (dashed) lines represent negative pathways, and blue (solid) lines represent positive pathways. For the optimized model (B), standardized coefficient estimates with ‘*’ represent statistically significant coefficients, non‐significant pathways were only retained if they represented biologically relevant relationships.

Specifically, for the top‐down model, we hypothesized that wolf occurrence would have a strong negative causal pathway to wild boar, as previous research in Eastern Europe and specifically the Romanian Carpathians has shown this to be a primary prey item in wolf diet (Rigg and Gorman 2004; Valdmann et al. 2005; Mori et al. 2016; Sin et al. 2019; Mysłajek et al. 2022); we also predicted negative causal pathways from wolves to red deer and roe deer, which have been identified as secondary prey items in the study area and predominant prey items for wolves elsewhere in Eastern Europe (Rigg and Gorman 2004; Nowak et al. 2005; Mori et al. 2016; Sin et al. 2019; Mysłajek et al. 2022). For lynx, we hypothesized negative causal pathways to roe deer and, to a lesser extent, hare; roe deer are the primary prey source for lynx throughout Eastern Europe (Jedrzejewski et al. 1996; Okarma et al. 1997; Herfindal et al. 2005; Valdmann et al. 2005; Molinari‐Jobin et al. 2007; Basille et al. 2009; Krofel et al. 2011) with hare and other small mammals as secondary prey items (Valdmann et al. 2005; Krofel et al. 2011). Although red deer fawns have been identified in lynx diet (Okarma et al. 1997), we did not hypothesize a negative causal pathway between lynx and red deer because our study does not overlap with the fawn season for red deer, and we expect environmental variables and interspecific interactions with wolves to be more important in determining the occurrence of red deer. Similarly, while red fox are known to prey on roe deer fawns, as the study period did not overlap with the fawn period for roe deer (Jarnemo 2004; Jarnemo and Liberg 2005), we did not include a negative pathway to roe deer. Previous research on interspecific interactions between apex carnivores (lynx and wolf) and wildcat in this system and similar systems found little to no evidence of interference competition (Wikenros et al. 2010; Dyck et al. 2022). However, a study in Slovenia and Italy documented wildcat scavenging on 38% of their experimentally set deer carcasses (Krofel et al. 2021). Similarly, a study in Poland found that red fox diet contained carcasses of wild boar and red deer killed by lynx or wolves (Jędrzejewski and Jędrzejewska 1992). Therefore, it is possible that there are positive relationships between apex predators and mesocarnivores due to the indirect supply of food through carcass remains. Wildcats and red fox are known to feed on rabbits and hares in other areas of Europe (Jędrzejewski and Jędrzejewska 1992; Malo et al. 2004) therefore, we predicted a negative causal pathway between both wildcat and red fox to hare. Given the similarity in the prey items of red fox and wildcat, we also included predicted possible competition between the two mesocarnivores with a negative causal pathway from the more common larger bodied red fox to wildcat. Finally, we also included causal pathways between environmental (exogenous) variables and both apex and mesocarnivores based on results of previous research in this system, which investigated the effect of *elevation*, *forest*, *road density*, and *TRI* on the marginal occupancy and co‐occurrence of lynx, wolf, and wildcat (Dyck et al. 2022). All hypothesized causal pathways for the top‐down model can be seen in Figure 2a.

#### 2.4.2 Bottom‐Up Processes

**FIGURE 3:**
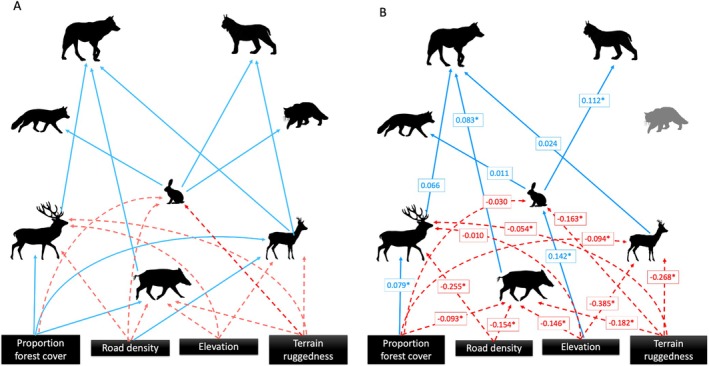
Hypothesized (A) and optimized (B) piecewise structural equation models (SEM) for bottom‐up species interaction and landscape characteristics in the Romanian Carpathians. Red (dashed) lines represent negative pathways, and blue (solid) lines represent positive pathways. For the optimized model (B), standardized coefficient estimates with ‘*’ represent statistically significant coefficients; non‐significant pathways were only retained if they represented biologically relevant relationships.

For the bottom‐up model, we hypothesized that prey availability and prey habitat would positively impact the occurrence of predators (directly and indirectly; Figure 3a). Prior research shows a high degree of spatiotemporal overlap between wolves and prey species; therefore, given previous literature on wolf diet in the region (see above), we predicted positive causal pathways from wild boar, red deer, and roe deer to wolves (Kittle et al. 2017; Rossa et al. 2021). Similarly, we predicted positive causal pathways from roe deer and hare to lynx (Basille et al. 2009; Filla et al. 2017). Environmental harshness (e.g., TRI and elevation) can have a negative effect on habitat selection of ungulates (although the degree varies among species); thus, we predicted negative causal pathways between TRI and elevation and the occurrence of ungulate species (Jiang et al. 2008; Zweifel‐Schielly et al. 2009; Heurich et al. 2015). Additionally, previous research found that 40%–50% forest cover was needed to support high densities of all three ungulate species in the Polish Carpathians (Borowik et al. 2013). Therefore, we predicted positive causal pathways between forest cover and ungulates. Research in the Carpathians and Spain found that red deer and wild boar respectively avoid roads (D'Amico et al. 2016; Bojarska et al. 2020), therefore we predicted a negative causal pathway between road density and both red deer and wild boar. However, research on the impacts of roads on roe deer vary. For example, D'Amico et al. (2016) roe deer in Spain avoided roads, while a study in France found that roe deer will use areas closer to roads depending on the time of day and habitat (Bonnot et al. 2013). Furthermore, research on white‐tailed deer in North America found a positive relationship between road density and deer abundance as possible benefits of roads (e.g., decreased predation, access to food resources) outweighed the cost of road mortality (Munro et al. 2012). Given that road mortality is low in the study area (most roads are low traffic or unpaved logging roads), we hypothesized a positive causal pathway between road density and roe deer in our study. Research on hares in Europe indicates that they prefer large open non‐fragmented areas with low road density (Roedenbeck and Voser 2008; Neumann et al. 2011; Mayer et al. 2023); thus, we also included negative causal pathways between road density, forest cover, and hare occurrence. Finally, based on knowledge of wildcat and red fox diet in Europe, we predicted positive causal pathways between hare and wildcat/red fox occurrence (Jędrzejewski and Jędrzejewska 1992; Malo et al. 2004; Soe et al. 2017), but no positive pathway from roe deer to red fox due to lack of overlap with the fawn season, which is a source of prey for red fox (Jarnemo 2004; Jarnemo and Liberg 2005).

### 2.5 Statistical Analysis

The structured equations in both models were initially fit with all hypothesized causal pathways described above (Figures 2 and 3). We then conducted tests of directed separation, a function that is built into the ‘peicewiseSEM’ package to assess independence claims (or missing pathways) among variables, to optimize each model (Lefcheck 2016; Stenegren et al. 2017). The tests of directed separation in the ‘peicewiseSEM’ package provides the significance (*p*‐value) for individual independence claims (Lefcheck 2016). Significant independence claims (*p* < 0.05) that were deemed biologically relevant (i.e., not just statistically significant, but representing plausible causal pathways) and fit the model hypotheses (i.e., top‐down or bottom‐up processes) were added to the model individually and evaluated based on goodness‐of‐fit using Fisher's *C* statistic. The Fisher's C statistic tests the fit of the given model to the data and is compared to a chi‐square distribution whereby a non‐significant chi‐square (*p* > 0.05) means that there is weak support for the sum of the conditional independence claims, and thus indicates the hypothesized relationships are consistent with the data (Lefcheck 2016). If an independence claim was significant and improved the model fit (lowered Fisher's *C*), then they were included in the optimized model (Stenegren et al. 2017). Significant independence claims that did not fit the model hypothesis or were not deemed biologically relevant were excluded. We also included bivariate (among two exogenous variables) correlated errors between species where we did not hypothesize a causal pathway if the independence claims were significant, indicating a correlation between the species likely due to underlying ecological processes, this removes them from the basis set and therefore they do not affect the goodness‐of‐fit tests (Lefcheck 2016). Additionally, any non‐significant claims that did not match the predicted or ecologically relevant direction of affect were removed from the model to reduce model complexity, hypothesized non‐significant claims were retained.

After implementing SEM analyses for top‐down and bottom‐up models separately, we developed a combined model by including only the retained paths from the two optimized models. The three final models were then evaluated using goodness‐of‐fit measures, Fisher's *C* and Chi square (*χ* ^2^) statistics and associated *p*‐values. For both goodness‐of‐fit measures, a lower statistic and a non‐significant *p*‐value (*p* > 0.05) indicate a good fitting model. We also used Nagelkerke pseudo *R* ^2^ values to evaluate model fit for each structured equation. The pseudo *R* ^2^ represents the proportion of explained variation of each structured equation relative to a null model (Nagelkerke 1991). The importance and direction of effect for individual paths was inferred by the standardized coefficient estimates (Std. estimate) that serve as a measure of effect size.

## 3 Results

We recorded a total of 3599 occurrences of our eight target species, with similar numbers of occurrences per season (45% winter and 55% autumn). Fox was detected the most in the study area (27% of occurrences; *n* = 985), followed by red deer (22%), wild boar (19%), roe deer (11%), lynx (9%), wolf (7%), wildcat (3%), and hare (2%).

### 3.1 SEM Models

The combined SEM model had the best model fit based on Fisher's *C* statistic and *χ* ^2^ (*C*_*28*_ = 27.973, *p* = 0.36; *χ* ^2^_23_ = 0, *p* = 1), compared with the optimized top‐down and bottom‐up models, which had significant *p*‐values for both goodness‐of‐fit tests, indicating poor model fit (Table 2). The worst fit model after optimization was the bottom‐up (*C*_*40*_ = 416.49, *p* = 0; *χ* ^2^_20_ = 345.09, *p* = 0). However, the optimized bottom‐up model was closer to our hypothesized bottom‐up model and contained pathways to all but one species (wildcat, Figure 3). The optimized top‐down model had a better fit than the bottom‐up model (*C*_*28*_ = 64.787, *p* = 0; *χ* ^2^_14_ = 42.531, *p* = 0), but after optimization it did not include three of the species of interest (red deer, roe deer, and wild boar), thus providing no support for top‐down processes acting on multiple trophic levels (Figure 2).

**TABLE 2:** Model comparison of piecewise structural equation models predicting causal pathways of species occurrence data from camera traps for Eurasian lynx ( *Lynx lynx* ), gray wolf ( *Canis lupus* ), European wildcat ( *Felis silvestris* ), red fox ( *Vulpes vulpes* ), European hare ( *Lepus europaeus* ), red deer ( *Cervus elaphus* ), roe deer ( *Capreolus capreolus* ), and wild boar ( *Sus scrofa* ) in the Romanian Carpathians.

Model	*C*	*p*	DF	*χ* ^2^	*p*	DF
Combined	27.973	0.36	26	0.00	1	23
Top‐down	64.787	0.00	28	42.531	0.00	14
Bottom‐up	416.49	0.00	40	345.09	0.00	20

*Note:* Model fit was evaluated using Fisher's *C* (*C*) and chi square (*χ* ^2^) statistics and associated *p*‐values (*p*) based on degrees of freedom (DF), where a significant *p*‐value (*p* < 0.05) indicates that the data structure is significantly different than that described by the model.

**FIGURE 4:**
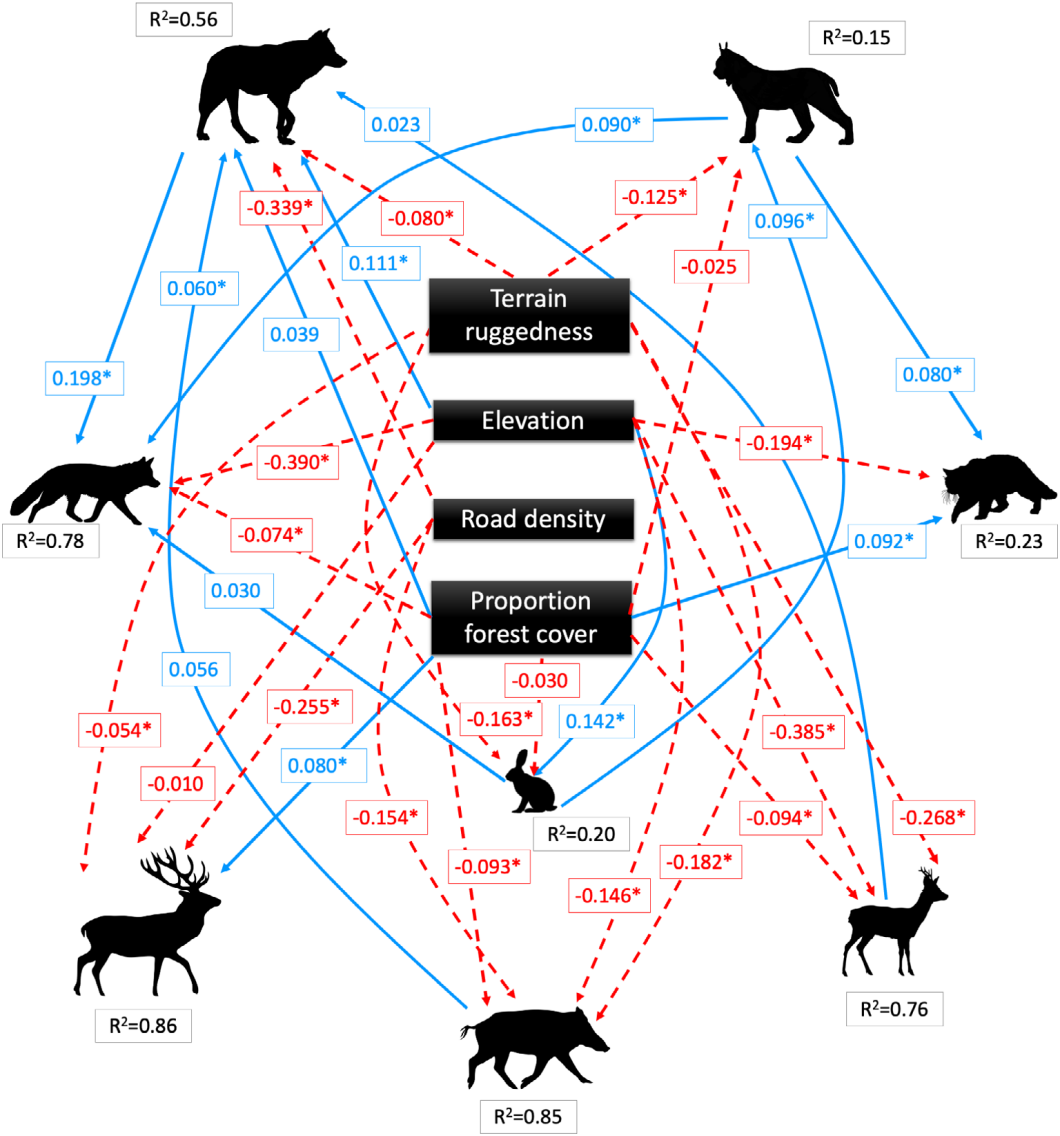
Optimized combined (including top‐down and bottom‐up processes) piecewise structural equation model (SEM) for species interaction and landscape characteristics in the Romanian Carpathians. Red (dashed) lines represent negative pathways, and blue (solid) lines represent positive pathways. Standardized coefficient estimates are presented in colored boxes corresponding to each path, estimates with ‘*’ represent statistically significant coefficients, non‐significant pathways were only retained if they represented biologically relevant relationships. Nagelkerke pseudo R_2_ values are presented for each species’ structured equation and represent the proportion of explained variation of each model relative to null models.

The individual pseudo R^2^ values for the combined model were high (Figure 4), indicating that the structured equations (GLMs) included in the network fit the data well, although we recognize the uncertainty surrounding the interpretation of these metrics (Smith and McKenna 2013), and most pathways included in our final combined model were significant with the direction of effect aligning with predictions (Table 3). The combined model contained many of our hypothesized pathways for landscape variables and bottom‐up processes (Figure 4); similarly to the optimized top‐down model, none of the top‐down causal pathways from carnivores to prey were included in the combined model. However, the combined model did include top‐down processes from apex carnivores to mesocarnivores, in the direction we hypothesized and based on the literature. Specifically, we found a positive effect between the occurrence of both apex carnivores and red fox (wolf:Std. estimate = 0.198, *p* < 0.0001; lynx: Std. estimate = 0.090, *p* = 0.001; Table 3) and between lynx and wildcat (Std. estimate = 0.080, *p* = 0.012; Table 3). Prey species occurrence had positive effects on three of the carnivores, wolf (wild boar: Std. estimate = 0.056, *p* = 0.064; roe deer: Std. estimate = 0.023, *p* = 0.578), lynx—hare (Std. estimate = 0.096, p = 0.001), and fox—hare (Std. estimate = 0.030, *p* < 0.385).

**TABLE 3:** Coefficient estimates, standard error (SE), degrees of freedom (DF), *p*‐value (*p*), and standardized coefficient estimates (Std. Estimate) for predictor variables included in the optimized piecewise structural equation model (SEM) predicting causal pathways of species occurrence data from camera traps for Eurasian lynx ( *Lynx lynx* ), gray wolf ( *Canis lupus* ), European wildcat ( *Felis silvestris* ), red fox ( *Vulpes vulpes* ), European hare ( *Lepus europaeus* ), red deer ( *Cervus elaphus* ), roe deer ( *Capreolus capreolus* ), and wild boar ( *Sus scrofa* ) in the Romanian Carpathians.

Response	Predictor	Estimate	SE	DF	*p*	Std. Estimate
Lynx	TRI	−0.174	0.059	134	0.003	−0.125
	Hare	0.098	0.030	134	0.001	0.096
	Forest	−0.035	0.054	134	0.514	−0.025
Wolf	Road density	−0.616	0.076	131	< 0.0001	−0.339
	Elevation	0.202	0.063	131	0.001	0.111
	TRI	−0.145	0.072	131	0.045	−0.080
	Wild boar	0.010	0.006	131	0.064	0.056
	Forest	0.071	0.068	131	0.300	0.039
	Roe deer	0.009	0.016	131	0.578	0.023
Fox	Elevation	−0.380	0.033	132	< 0.0001	−0.390
	Wolf	0.056	0.007	132	< 0.0001	0.198
	Lynx	0.027	0.009	132	0.001	0.090
	Forest	0.072	0.028	132	0.013	0.074
	Hare	0.022	0.025	132	0.385	0.030
Wildcat	Elevation	−0.467	0.099	134	< 0.0001	−0.194
	Forest	0.223	0.102	134	0.028	0.092
	Lynx	0.060	0.023	134	0.012	0.080
Hare	TRI	−0.533	0.151	134	< 0.001	−0.164
	Elevation	0.460	0.118	134	< 0.001	0.142
	Forest	−0.099	0.125	134	0.429	−0.030
Red deer	Road density	−0.528	0.042	133	< 0.0001	−0.255
	Forest	0.163	0.040	133	< 0.0001	0.079
	TRI	−0.112	0.038	133	0.003	−0.054
	Elevation	−0.021	0.035	133	0.537	−0.010
Roe deer	Elevation	−0.486	0.054	134	< 0.0001	−0.385
	TRI	−0.338	0.057	134	< 0.0001	−0.268
	Forest	−0.119	0.041	134	0.003	−0.094
Wild boar	TRI	−0.314	0.042	133	< 0.0001	−0.182
	Road density	−0.264	0.040	133	< 0.0001	−0.154
	Elevation	−0.251	0.039	133	< 0.0001	−0.146
	Forest	−0.159	0.030	133	< 0.0001	−0.093

*Note:* Predictors for each species are ordered from most to least important based on the Std. Estimate which is a measure of effect size.

Overall, landscape variables played a significant role in explaining variation in species occurrence, with more pathways and greater effect size per pathway compared to interspecific interactions (Figure 4 and Table 3). For example, three landscape variables (TRI, elevation, and road density) had the single greatest effect size for all eight species (Table 3). Specifically, TRI had direct negative effects on all prey species and direct or indirect negative effects on all predator species. Similarly, elevation also had direct or indirect effects on all species, although some relationships were positive while others were negative. Elevation positively affected wolf and hare occurrence, while negatively impacting fox, wildcat, wild boar, roe deer, and red deer directly, and wolves and foxes indirectly through wild boar and roe deer, respectively. Road density negatively affected the occurrence of wolves, red deer, and wild boar, with an additional indirect effect on wolves mediated through wild boar (Figure 4 and Table 3). Finally, although not a top predictor for any species, forest cover was retained as a predictor for all species, with positive effects on red deer, wildcat, fox, and wolves, and negative effects on wild boar, roe deer, hare, and lynx.

## 4 Discussion

### 4.1 Implications for Carnivore Recovery and Rewilding

Results from our study indicate that while species interactions play a role in shaping community dynamics, landscape characteristics have a greater effect on species abundance and distribution in this Romanian Carpathian system. This highlights the fact that suitable habitats and environmental conditions with low human impact and a high degree of natural integrity are a pre‐requisite for maintaining viable mammalian populations and communities. Introducing influential species, such as apex predators, for trophic rewilding efforts alone is likely not sufficient to restore ecosystem function, but rather should be paired with efforts to preserve wildlife habitat. For example, results from the SEM models indicate that while apex predators are known to shape ecosystems through top‐down regulation, in this system their impact on prey species is not highly noticeable compared to the potential for landscape characteristics and prey species to regulate predator abundance (within the autumn and winter seasons analyzed here). While we found significant relationships between apex carnivores and mesocarnivores, these were relatively weak compared to environmental influences and did not cascade through trophic levels to prey species.

The effect of apex carnivores on mesocarnivores was positive, indicating that with sufficient resource availability, rewilding and recolonization of apex carnivores would likely not lead to long‐term declines in existing mesocarnivore populations. We expect the positive relationships between apex carnivores and mesocarnivores are due to supplemental food supplied by carcasses of lynx and wolf kills; for example, fox and wildcat have been observed scavenging on wolf and lynx kills in Central Europe (Jędrzejewski and Jędrzejewska 1992; Krofel et al. 2021). These results also support previous research in this system, which found that co‐occupancy of wolf, lynx, and wildcat was high and conditional occupancy (occupancy of one species contingent on the presence or absence of another) was higher for all three species when another species was present (Dyck et al. 2022).

We found evidence for bottom‐up effects from prey species to carnivores (hare—lynx, wild boar—wolf, roe deer—wolf, and hare—fox), but several were relatively weak (*p* > 0.05) and they were generally weaker than landscape variables (Figure 4 and Table 3). The Romanian Carpathians are home to large stable prey populations, and this likely contributed to a lower effect size of prey on carnivores than predicted. However, the presence of bottom‐up processes does emphasize the need for an adequate prey base to support viable carnivore populations (Breitenmoser 1998). This is relevant given wolf recolonization and the popularity of trophy hunting (wild boar and red deer) in Europe (Soliño et al. 2017). As hunting and predation by wolves are likely to have compounding effects on prey populations, adequate measures would need to be taken in areas with wolf or lynx recolonization or if reintroductions occur to ensure adequate prey availability (Linnell and Cretois 2018). In fact, given the challenges in regulating overabundant ungulate populations in many areas of Europe without apex predators (Valente et al. 2020; Carpio et al. 2021), wolf and lynx recovery can alleviate human‐wildlife conflict in the context of ungulates (e.g., crop raiding, vehicle collisions, disease) (Linnell et al. 2020). Contrastingly, we did not find expected bottom‐up or top‐down effects between lynx and roe deer, and instead found only a positive effect of hare on lynx. Efforts to reintroduce lynx to Scotland (Johnson and Greenwood 2020) have been marred by concerns about the effects of lynx on deer populations, alongside increasing human‐wildlife conflict (Drouilly and O'Riain 2021). Our results indicate that potential effects of lynx on deer may be limited if hares are present in the landscape, as lynx distribution and abundance appear to be influenced by their smaller prey.

### 4.2 Influence of Landscape Characteristics on Predators and Prey

This study provides insights into specific habitat requirements that are needed to support prey and predator populations during the cold season in this Eastern European system. Elevation and TRI had direct negative effects on the occurrence of most prey species (with the exception hare [positive] and red deer [no significant effect] for elevation), indicating the need to preserve areas of less topographically fragmented habitat at moderate to low elevation. These findings are supported by previous research on herbivores in Europe; red and roe deer are known to move to less fragmented areas connected to their lower winter grounds after the rut season (November—December) (Zweifel‐Schielly et al. 2009) and roe deer prefer lower elevation areas with less snow (Jiang et al. 2008; Heurich et al. 2015). Elevation and TRI also had negative effects on mesocarnivores and apex carnivores respectively, further highlighting the importance of connected and less topographically fragmented habitat at moderate to low elevation. These results are also supported by previous research on species occupancy whereby elevation and TRI had negative effects on the marginal occupancy of wildcat and wolf respectively (Dyck et al. 2022). Forest cover had mixed effects on species occurrence, with direct positive effects on wolf, wildcat, and red deer and negative effects on wild boar, roe deer, and lynx. We predicted positive effects of forest cover on all ungulate species based on previous research (Borowik et al. 2013), which indicated that 40% – 50% forest cover was needed to support populations of red deer, roe deer, and wild boar. However, our study area is heavily forested (mean = 77%) and thus forest cover is likely not a limitation; instead, the need for overwintering ranges and open habitat with high‐quality resources may be more limiting in this area (Bonnot et al. 2013; Padié et al. 2015). Lastly, as predicted, road density had a negative effect on wolf, red deer, and wild boar which further emphasizes the need for preserving contiguous habitat. Despite previous research finding positive relationships between road density and both lynx and roe deer (Bonnot et al. 2013; Dyck et al. 2022), our optimized SEM did not include effects of road density to these species. Studies on roe deer have found conflicting results on the effects of roads (Bonnot et al. 2013; D'Amico et al. 2016) and therefore our findings are not surprising. However, for lynx, Dyck et al. (2022) found a positive relationship between road density and lynx occupancy for only the winter season and not autumn; therefore, it is possible that roads only benefit lynx during winter when snow is deepest allowing for easier movement; however, when fall and winter seasons were combined, as in our analysis, the effect of road density may be diluted.

### 4.3 Study Constraints and Limitations

One of the main limitations of our work is that it only encompassed two primarily‐cold seasons (autumn and winter) due to the initial camera design to address questions about lynx density (Iosif et al. 2022). It is possible that hypothesized pathways that were not retained in our SEM could be relevant in other seasons (i.e., spring/summer) and vice versa. As such, investigating the effects of seasonality on the interactions between carnivores and their prey in this system using longer‐term datasets and adding additional species is warranted. In particular, brown bears are absent from our analysis due to the overlap between the study period and the hibernation season, and we expect that brown bears will have variable effects on the mammalian assemblage. For example, brown bear density can affect the establishment of new wolf packs in Scandinavia through competitive interactions (Ordiz et al. 2015), and brown bears can contribute to the loss of Eurasian lynx prey biomass via kleptoparasitism (Krofel et al. 2012). At the same time, we expect that the effect of brown bears on wild ungulates to be relatively modest, as wild ungulates make up only ~10% of brown bear diet in Eurasia (Niedziałkowska et al. 2019). Another species absent from our study is the European bison, an ecosystem engineer species, which was extirpated from Romania > 100 years ago. Ongoing rewilding efforts resulted in the establishment of several populations in the Romanian Carpathians (Tănăsescu 2019; Dănilă et al. 2022). One new population has been established in the study area after this camera trapping study started (Holland 2024; R. Iosif, pers. comm.) and there is anecdotal evidence of European bison predation by wolves and brown bears. Future camera trapping studies could provide the opportunity to elucidate the role of European bison in the Carpathian mammalian assemblage.

Furthermore, our study was focused on predator–prey dynamics of large‐bodied mammals and excluded many small mammals, birds, and invertebrates, which are hard to reliably detect with camera traps. Our landscape variables are extracted at a relatively coarse level, and fine‐scale data on primary productivity or disturbances such as forestry operations would provide additional information for the models. Ideally, long‐term studies that expand to the broader ecological community are needed to fully contextualize the dynamics in this system. However, long‐term community‐level studies are difficult to fund and maintain; many (non‐tropical) long‐term food web studies are often located in areas with low productivity (Arctic, boreal forest; e.g., Krebs et al. 2023; Gauthier et al. 2024). Community complexity increases with increased productivity, and the trophic relationships and interspecific interactions vary with latitude and regional context (e.g., see review of range‐wide Eurasian lynx diet; Khorozyan and Heurich 2023). While our study only provides a snapshot into the complex interactions between apex carnivores, mesocarnivores, and prey species, the focus on a high‐productivity system that harbors the most intact assemblage of mammalian species in Europe is valuable for understanding how mammalian communities at equilibrium are functioning in the context of rewilding. Both reintroductions and natural expansions of large carnivores are subject to debate due to increased human‐wildlife conflict, and some conflict stems from the perceived notion that predators would lead to declines in valuable game species (Linnell and Cretois 2018). Our research suggests that in systems at equilibrium, viable populations of prey species can likely support the needs of both hunters and predators. Lastly, our research shows that interactions within mammalian assemblages are complex, and interaction pathways identified in other systems may not be generalizable. As such, conservation efforts (including trophic rewilding) should not assume that top‐down or bottom‐up processes will follow assumed pathways, but rather that they are shaped by the local landscape and human impact context.

The study findings have implications for current conservation efforts in Europe and globally, emphasizing the need for management and conservation strategies that incorporate considerations of landscape processes structuring ecological communities and interspecific interactions. Moreover, the study highlights the critical importance of preserving adequate natural habitats, underscoring that effective species conservation must go hand in hand with conserving natural spaces.

## Author Contributions

**Marissa A. Dyck:** conceptualization (equal), data curation (equal), formal analysis (lead), methodology (equal), visualization (lead), writing – original draft (lead), writing – review and editing (equal). **Ruben Iosif:** conceptualization (supporting), data curation (lead), methodology (equal), project administration (lead), resources (equal), writing – review and editing (equal). **Barbara Promberger–Fürpass:** data curation (equal), funding acquisition (lead), methodology (equal), project administration (equal), resources (lead), writing – review and editing (equal). **Viorel D. Popescu:** conceptualization (equal), data curation (equal), formal analysis (supporting), funding acquisition (lead), methodology (equal), project administration (equal), resources (equal), supervision (lead), writing – original draft (supporting), writing – review and editing (equal).

## Disclosure

Statement on inclusion: Our study brings together authors from different countries, including scientists based in the country where the study was carried out. All authors were engaged early on with the research and study design to ensure that the diverse sets of perspectives they represent were considered from the onset. Whenever relevant, literature published by scientists from the region was cited; efforts were made to consider relevant work conducted in the country and work published in the local language.

## Conflicts of Interest

The authors declare no conflicts of interest.

## Data Availability

Data and code is available on GitHub (https://github.com/marissadyck/Romania_SEM) and will be made available via Dryad Digital Repository after manuscript acceptance. A copy of the analysis printout was submitted with the manusscript.

## References

[ece371381-bib-0001] Allen, D. C. , C. C. Vaughn , J. F. Kelly , J. T. Cooper , and M. H. Engel . 2012. “Bottom‐Up Biodiversity Effects Increase Resource Subsidy Flux Between Ecosystems.” Ecology 93, no. 10: 2165–2174. 10.1890/11-1541.1. 23185878

[ece371381-bib-0002] Basille, M. , I. Herfindal , H. Santin‐Janin , et al. 2009. “What Shapes Eurasian Lynx Distribution in Human Dominated Landscapes: Selecting Prey or Avoiding People?” Ecography 32, no. 4: 683–691. 10.1111/j.1600-0587.2009.05712.x.

[ece371381-bib-0003] Berger, J. , J. E. Swenson , and I.‐L. Persson . 2001. “Recolonizing Carnivores and Naïve Prey: Conservation Lessons From Pleistocene Extinctions.” Science 291, no. 5506: 1036–1039. 10.1126/science.1056466. 11161215

[ece371381-bib-0004] Blüthgen, N. , N. Stork , and K. Fiedler . 2004. “Bottom‐Up Control and Co‐Occurrence in Complex Communities: Honeydew and Nectar Determine a Rainforest Ant Mosaic.” Oikos 106, no. 2: 344–358. 10.1111/j.0030-1299.2004.12687.x.

[ece371381-bib-0005] Boitani, L. 2000. “Action Plan for the Conservation of the Wolves (*Canis lupus*) in Europe. Convention on the Conservation of European Wildlife and Natural Habitats (Bern Convention) Nature and Environment, No. 113 Council of Europe Publishing, 2000, Council of Europe Publishing.”

[ece371381-bib-0006] Boitani, L. , and J. D. Linnell . 2015. “Bringing Large Mammals Back: Large Carnivores in Europe.” In Rewilding European Landscapes, edited by H. M. Pereira and L. M. Navarro , 67–84. SpringerOpen.

[ece371381-bib-0007] Bojarska, K. , K. Kurek , S. Śnieżko , et al. 2020. “Winter Severity and Anthropogenic Factors Affect Spatial Behaviour of Red Deer in the Carpathians.” Mammal Research 65, no. 4: 815–823. 10.1007/s13364-020-00520-z.

[ece371381-bib-0008] Bonnot, N. , N. Morellet , H. Verheyden , et al. 2013. “Habitat Use Under Predation Risk: Hunting, Roads and Human Dwellings Influence the Spatial Behaviour of Roe Deer.” European Journal of Wildlife Research 59, no. 2: 185–193. 10.1007/s10344-012-0665-8.

[ece371381-bib-0009] Borowik, T. , T. Cornulier , and B. Jędrzejewska . 2013. “Environmental Factors Shaping Ungulate Abundances in Poland.” Acta Theriologica 58, no. 4: 403–413. 10.1007/s13364-013-0153-x. 24244044 PMC3786087

[ece371381-bib-0010] Bowman, J. , G. Forbes , and T. Dilworth . 2001. “Landscape Context and Small‐Mammal Abundance in a Managed Forest.” Forest Ecology and Management 140, no. 2: 249–255. 10.1016/S0378-1127(00)00315-7.

[ece371381-bib-0011] Breitenmoser, U. 1998. “Large Predators in the Alps: The Fall and Rise of Man's Competitors.” Biological Conservation 83, no. 3: 279–289. 10.1016/S0006-3207(97)00084-0.

[ece371381-bib-0012] Breitenmoser, U. 2000. “Action Plan for the Conservation of the Eurasian Lynx in Europe (*Lynx lynx*).”

[ece371381-bib-0013] Burkepile, D. E. , C. E. Burns , C. J. Tambling , et al. 2013. “Habitat Selection by Large Herbivores in a Southern African Savanna: The Relative Roles of Bottom‐Up and Top‐Down Forces.” Ecosphere 4, no. 11: art139. 10.1890/ES13-00078.1.

[ece371381-bib-0014] Cano‐Martínez, R. , N. H. Thorsen , T. R. Hofmeester , et al. 2024. “Bottom‐Up Rather Than Top‐Down Mechanisms Determine Mesocarnivore Interactions in Norway.” Ecology and Evolution 14, no. 3: e11064. 10.1002/ece3.11064. 38463636 PMC10920318

[ece371381-bib-0015] Carpio, A. J. , M. Apollonio , and P. Acevedo . 2021. “Wild Ungulate Overabundance in Europe: Contexts, Causes, Monitoring and Management Recommendations.” Mammal Review 51, no. 1: 95–108. 10.1111/mam.12221.

[ece371381-bib-0016] Chapron, G. , P. Kaczensky , J. D. C. Linnell , et al. 2014. “Recovery of Large Carnivores in Europe's Modern Human‐Dominated Landscapes.” Science 346, no. 6216: 1517–1519. 10.1126/science.1257553. 25525247

[ece371381-bib-1003] Connell, J. H. 1983. “On the Prevalence and Relative Importance of Interspecific Competition: Evidence From Field Experiments.” American Naturalist 122, no. 5: 661–696.

[ece371381-bib-0017] Cromsigt, J. P. G. M. , M. te Beest , G. I. H. Kerley , M. Landman , E. le Roux , and F. A. Smith . 2018. “Trophic Rewilding as a Climate Change Mitigation Strategy?” Philosophical Transactions of the Royal Society, B: Biological Sciences 373, no. 1761: 20170440. 10.1098/rstb.2017.0440. PMC6231077 30348867

[ece371381-bib-0018] Crooks, K. R. , and M. E. Soulé . 1999. “Mesopredator Release and Avifaunal Extinctions in a Fragmented System.” Nature 400, no. 6744: 563–566. 10.1038/23028.

[ece371381-bib-0019] D'Amico, M. , S. Périquet , J. Román , and E. Revilla . 2016. “Road Avoidance Responses Determine the Impact of Heterogeneous Road Networks at a Regional Scale.” Journal of Applied Ecology 53, no. 1: 181–190. 10.1111/1365-2664.12572.

[ece371381-bib-0020] Dănilă, G. , S. Cătănoiu , V. Simioniuc , and S. Roșca . 2022. “The Reintroduction Analysis of European Bison ( *Bison bonasus* L., 1758) in the North of Romania and the Identification of the Most Favourable Locations.” Forests 13, no. 6: 920. 10.3390/f13060920.

[ece371381-bib-0021] Davoli, M. , A. Ghoddousi , F. M. Sabatini , E. Fabbri , R. Caniglia , and T. Kuemmerle . 2022. “Changing Patterns of Conflict Between Humans, Carnivores and Crop‐Raiding Prey as Large Carnivores Recolonize Human‐Dominated Landscapes.” Biological Conservation 269: 109553. 10.1016/j.biocon.2022.109553.

[ece371381-bib-0022] Dorresteijn, I. , J. Schultner , D. G. Nimmo , et al. 2015. “Incorporating Anthropogenic Effects Into Trophic Ecology: Predator–Prey Interactions in a Human‐Dominated Landscape.” Proceedings of the Royal Society 282, no. 8: 1814. 10.1098/rspb.2015.1602. PMC4571711 26336169

[ece371381-bib-0023] Drouilly, M. , and M. J. O'Riain . 2021. “Rewilding the World's Large Carnivores Without Neglecting the Human Dimension: A Response to Reintroducing the Eurasian Lynx to Southern Scotland, England and Wales.” Biodiversity and Conservation 30, no. 3: 917–923. 10.1007/s10531-021-02112-y.

[ece371381-bib-0024] Dyck, M. A. , R. Iosif , B. Promberger–Fürpass , and V. D. Popescu . 2022. “Dracula's Ménagerie: A Multispecies Occupancy Analysis of Lynx, Wildcat, and Wolf in the Romanian Carpathians.” Ecology and Evolution 12, no. 5: e8921. 10.1002/ece3.8921. 35600678 PMC9109232

[ece371381-bib-0025] Elmhagen, B. , and S. P. Rushton . 2007. “Trophic Control of Mesopredators in Terrestrial Ecosystems: Top‐Down or Bottom‐Up?” Ecology Letters 10, no. 3: 197–206. 10.1111/j.1461-0248.2006.01010.x. 17305803

[ece371381-bib-0026] Filla, M. , J. Premier , N. Magg , et al. 2017. “Habitat Selection by Eurasian Lynx ( *Lynx lynx* ) is Primarily Driven by Avoidance of Human Activity During Day and Prey Availability During Night.” Ecology and Evolution 7, no. 16: 6367–6381. 10.1002/ece3.3204. 28861240 PMC5574813

[ece371381-bib-0027] Frederiksen, M. , M. Edwards , A. J. Richardson , N. C. Halliday , and S. Wanless . 2006. “From Plankton to Top Predators: Bottom‐Up Control of a Marine Food Web Across Four Trophic Levels.” Journal of Animal Ecology 75, no. 6: 1259–1268. 10.1111/j.1365-2656.2006.01148.x. 17032358

[ece371381-bib-0028] Gauthier, G. , D. Berteaux , J. Bêty , et al. 2024. “Scientific Contributions and Lessons Learned From 30 Years of Ecological Monitoring of the Bylot Island Tundra Ecosystem.” Frontiers in Ecology and Evolution 12: 1359745. 10.3389/fevo.2024.1359745.

[ece371381-bib-0029] Gigliotti, L. C. , R. Slotow , L. T. B. Hunter , J. Fattebert , C. Sholto‐Douglas , and D. S. Jachowski . 2020. “Context Dependency of Top‐Down, Bottom‐Up and Density‐Dependent Influences on Cheetah Demography.” Journal of Animal Ecology 89, no. 2: 449–459. 10.1111/1365-2656.13099. 31469173

[ece371381-bib-0030] Hebblewhite, M. , C. A. White , C. G. Nietvelt , et al. 2005. “Human Activity Mediates a Trophic Cascade Caused by Wolves.” Ecology 86, no. 8: 2135–2144. 10.1890/04-1269.

[ece371381-bib-0031] Herfindal, I. , J. D. C. Linnell , J. Odden , E. B. Nilsen , and R. Andersen . 2005. “Prey Density, Environmental Productivity and Home‐Range Size in the Eurasian Lynx ( *Lynx lynx* ).” Journal of Zoology 265, no. 1: 63–71. 10.1017/S0952836904006053.

[ece371381-bib-0032] Heurich, M. , T. T. G. Brand , M. Y. Kaandorp , P. Šustr , J. Müller , and B. Reineking . 2015. “Country, Cover or Protection: What Shapes the Distribution of Red Deer and Roe Deer in the Bohemian Forest Ecosystem?” PLoS One 10, no. 3: e0120960. 10.1371/journal.pone.0120960. 25781942 PMC4363369

[ece371381-bib-0114] Holland, L. C. V. 2024. “Spatial Ecology and Habitat Selection of Translocated European Bison in the Romanian Carpathians.” Master Thesis, Columbia University, Department of Ecology, Evolution and Environmental Biology, New York, NY, USA.

[ece371381-bib-0033] Iosif, R. , V. D. Popescu , L. Ungureanu , C. Șerban , M. A. Dyck , and B. Promberger‐Fürpass . 2022. “Eurasian Lynx Density and Habitat Use in One of Europe's Strongholds, the Romanian Carpathians.” Journal of Mammalogy 103, no. 2: gyab157. 10.1093/jmammal/gyab157.

[ece371381-bib-0034] Iosif, R. , T. Skrbinšek , M. Jelencic , et al. 2021. “FCC Report on Monitoring Brown Bears Using Non‐Invasive DNA Samping in the Romanian Carpathians.” https://www.carpathia.org/wp‐content/uploads/2021/10/FCC‐Report‐on‐monitoring‐brown‐bear‐using‐non‐invasive‐DNA‐sampling‐in‐the‐Romanian‐Carpathians.pdf.

[ece371381-bib-0035] Jarnemo, A. 2004. “Predation Processes: Behavioural Interactions Between Red Fox and Roe Deer During the Fawning Season.” Journal of Ethology 22, no. 2: 167–173. 10.1007/s10164-004-0118-2.

[ece371381-bib-0036] Jarnemo, A. , and O. Liberg . 2005. “Red Fox Removal and Roe Deer Fawn Survival—A 14‐Year Study.” Journal of Wildlife Management 69, no. 3: 1090–1098. 10.2193/0022-541X(2005)069[1090:RFRARD]2.0.CO;2.

[ece371381-bib-0037] Jędrzejewski, W. , and B. Jędrzejewska . 1992. “Foraging and Diet of the Red Fox *Vulpes vulpes* in Relation to Variable Food Resources in Biatowieza National Park, Poland.” Ecography 15, no. 2: 212–220. 10.1111/j.1600-0587.1992.tb00027.x.

[ece371381-bib-0038] Jedrzejewski, W. , B. Jedrzejewska , H. Okarma , K. Schmidt , A. N. Bunevich , and L. Milkowski . 1996. “Population Dynamics (1869–1994), Demography, and Home Ranges of the Lynx in Bialowieza Primeval Forest (Poland and Belarus).” Ecography 19, no. 2: 122–138. 10.1111/j.1600-0587.1996.tb00163.x.

[ece371381-bib-0039] Jiang, G. , M. Zhang , and J. Ma . 2008. “Habitat Use and Separation Between Red Deer Cervus Elaphus Xanthopygus and Roe Deer *Capreolus Pygargus Bedfordi* in Relation to Human Disturbance in the Wandashan Mountains, Northeastern China.” Wildlife Biology 14, no. 1: 92–100. 10.2981/0909-6396(2008)14[92:HUASBR]2.0.CO;2.

[ece371381-bib-0040] Johnson, R. , and S. Greenwood . 2020. “Assessing the Ecological Feasibility of Reintroducing the Eurasian Lynx (*Lynx Lynx*) to Southern Scotland, England and Wales.” Biodiversity and Conservation 29, no. 3: 771–797. 10.1007/s10531-019-01909-2.

[ece371381-bib-0041] Karimi, L. , and D. Meyer . 2014. “Structural Equation Modeling in Psychology: The History, Development and Current Challenges.” International Journal of Psychological Studies 6, no. 4: 123. 10.5539/ijps.v6n4p123.

[ece371381-bib-0042] Keeler, M. S. , F. S. Chew , B. C. Goodale , and J. M. Reed . 2006. “Modelling the Impacts of Two Exotic Invasive Species on a Native Butterfly: Top‐Down vs. Bottom‐Up Effects.” Journal of Animal Ecology 75, no. 3: 777–788. 10.1111/j.1365-2656.2006.01098.x. 16689960

[ece371381-bib-0043] Khorozyan, I. , and M. Heurich . 2023. “Patterns of Predation by the Eurasian *Lynx Lynx Lynx* Throughout Its Range: Ecological and Conservation Implications.” Mammal Review 53, no. 3: 177–188. 10.1111/mam.12317.

[ece371381-bib-0044] Kittle, A. M. , M. Anderson , T. Avgar , et al. 2017. “Landscape‐Level Wolf Space Use Is Correlated With Prey Abundance, Ease of Mobility, and the Distribution of Prey Habitat.” Ecosphere 8, no. 4: e01783. 10.1002/ecs2.1783.

[ece371381-bib-0045] Kohl, M. T. , D. R. Stahler , M. C. Metz , et al. 2018. “Diel Predator Activity Drives a Dynamic Landscape of Fear.” Ecological Monographs 88, no. 4: 638–652. 10.1002/ecm.1313.

[ece371381-bib-0046] Krebs, C. J. , S. Boutin , R. Boonstra , et al. 2023. “Long‐Term Monitoring in the Boreal Forest Reveals High Spatio‐Temporal Variability Among Primary Ecosystem Constituents.” Frontiers in Ecology and Evolution 11: 1187222. 10.3389/fevo.2023.1187222.

[ece371381-bib-0047] Krofel, M. , D. Huber , and I. Kos . 2011. “Diet of Eurasian *lynx Lynx lynx* in the Northern Dinaric Mountains (Slovenia and Croatia).” Acta Theriologica 56, no. 4: 315–322. 10.1007/s13364-011-0032-2.

[ece371381-bib-0048] Krofel, M. , D. Južnič , and M. L. Allen . 2021. “Scavenging and Carcass Caching Behavior by European Wildcat ( *Felis silvestris* ).” Ecological Research 36, no. 3: 556–561. 10.1111/1440-1703.12211.

[ece371381-bib-0049] Krofel, M. , I. Kos , and K. Jerina . 2012. “The Noble Cats and the Big Bad Scavengers: Effects of Dominant Scavengers on Solitary Predators.” Behavioral Ecology and Sociobiology 66, no. 9: 1297–1304. 10.1007/s00265-012-1384-6.

[ece371381-bib-0050] Kuijper, D. P. J. , D. P. Kuijper , E. Sahlén , et al. 2016. “Paws Without Claws? Ecological Effects of Large Carnivores in Anthropogenic Landscapes.” Proceedings of the Biological Sciences 283, no. 1841: 1–9. 10.1098/rspb.2016.1625. PMC5095381 27798302

[ece371381-bib-0051] Landa, A. , M. Lindén , and I. Kojola . 2000. “Action Plan for the Conservation of Wolverines (*Gulo gulo*) in Europe.” Nature and Environment, No. 115. Council of Europe Publishing.

[ece371381-bib-0052] Lefcheck, J. S. 2016. “piecewiseSEM: Piecewise Structural Equation Modelling in r for Ecology, Evolution, and Systematics.” Methods in Ecology and Evolution 7, no. 5: 573–579. 10.1111/2041-210X.12512.

[ece371381-bib-0053] Letnic, M. , F. Koch , C. Gordon , M. S. Crowther , and C. R. Dickman . 2009. “Keystone Effects of an Alien Top‐Predator Stem Extinctions of Native Mammals.” Proceedings of the Royal Society B: Biological Sciences 276, no. 1671: 3249–3256. 10.1098/rspb.2009.0574. PMC2817164 19535372

[ece371381-bib-0054] Levi, T. , and C. C. Wilmers . 2012. “Wolves–coyotes–foxes: a cascade among carnivores.” Ecology 93, no. 4: 921–929. 10.1890/11-0165.1. 22690642

[ece371381-bib-0055] Linnell, J. D. , J. D. C. Linnell , B. Cretois , et al. 2020. “The Challenges and Opportunities of Coexisting With Wild Ungulates in the Human‐Dominated Landscapes of Europe's Anthropocene.” Biological Conservation 244: 108500. 10.1016/j.biocon.2020.108500.

[ece371381-bib-0056] Linnell, J. D. C. , and B. Cretois . 2018. “Research for AGRI Committee—The Revival of Wolves and Other Large Predators and its Impact on Farmers and their Livelihood in Rural Regions of Europe. Belgium: EPRS: European Parliamentary Research Service.” https://policycommons.net/artifacts/1332425/research‐for‐agri‐committee/1935982/.

[ece371381-bib-0058] Madin, E. M. , J. S. Madin , and D. J. Booth . 2011. “Landscape of Fear Visible From Space.” Scientific Reports 1, no. 1: 1–4. 10.1038/srep00014. 22355533 PMC3216502

[ece371381-bib-0059] Malo, A. F. , J. Lozano , D. L. Huertas , and E. Virgós . 2004. “A Change of Diet From Rodents to Rabbits ( *Oryctolagus cuniculus* ). Is the Wildcat ( *Felis silvestris* ) a Specialist Predator?” Journal of Zoology 263, no. 4: 401–407. 10.1017/S0952836904005448.

[ece371381-bib-0060] Matassa, C. M. , and G. C. Trussell . 2011. “Landscape of Fear Influences the Relative Importance of Consumptive and Nonconsumptive Predator Effects.” Ecology 92, no. 12: 2258–2266. 10.1890/11-0424.1. 22352165

[ece371381-bib-0061] Mayer, M. , C. Fischer , N. Blaum , P. Sunde , and W. Ullmann . 2023. “Influence of Roads on Space Use by European Hares in Different Landscapes.” Landscape Ecology 38, no. 1: 131–146. 10.1007/s10980-022-01552-3.

[ece371381-bib-0062] Molinari‐Jobin, A. , F. Zimmermann , A. Ryser , et al. 2007. “Variation in Diet, Prey Selectivity and Home‐Range Size of Eurasian *Lynx Lynx Lynx* in Switzerland.” Wildlife Biology 13, no. 4: 393–405. 10.2981/0909-6396(2007)13[393:VIDPSA]2.0.CO;2.

[ece371381-bib-0063] Mori, E. , L. Benatti , S. Lovari , and F. Ferretti . 2016. “What Does the Wild Boar Mean to the Wolf?” European Journal of Wildlife Research 63, no. 1: 9. 10.1007/s10344-016-1060-7.

[ece371381-bib-0064] Munro, K. G. , J. Bowman , and L. Fahrig . 2012. “Effect of Paved Road Density on Abundance of White‐Tailed Deer.” Wildlife Research 39, no. 6: 478. 10.1071/WR11152.

[ece371381-bib-0065] Mysłajek, R. W. , P. Stachyra , M. Figura , et al. 2022. “Diet of the Grey Wolf *Canis Lupus* in Roztocze and Solska Forest, South‐East Poland.” Journal of Vertebrate Biology 71: 22040. 10.25225/jvb.22040.

[ece371381-bib-0066] Nagelkerke, N. J. D. 1991. “Miscellanea A Note on a General Definition of the Coefficient of Determination.” Biometrika 78, no. 3: 691–692.

[ece371381-bib-0067] Neumann, F. , S. Schai‐Braun , D. Weber , and V. Amrhein . 2011. “European Hares Select Resting Places for Providing Cover.” Hystrix, The Italian Journal of Mammalogy 22, no. 2. 10.4404/hystrix-22.2-4546.

[ece371381-bib-0068] Niedziałkowska, M. , M. W. Hayward , T. Borowik , W. Jędrzejewski , and B. Jędrzejewska . 2019. “A Meta‐Analysis of Ungulate Predation and Prey Selection by the Brown Bear *Ursus arctos* in Eurasia.” Mammal Research 64, no. 1: 1–9. 10.1007/s13364-018-0396-7.

[ece371381-bib-0069] Nowak, S. , R. W. Mysłajek , and B. Jędrzejewska . 2005. “Patterns of Wolf *canis lupus* Predation on Wild and Domestic Ungulates in the Western Carpathian Mountains (S Poland).” Acta Theriologica 50, no. 2: 263–276. 10.1007/BF03194489.

[ece371381-bib-0070] Okarma, H. , W. Jedrzejewski , K. Schmidt , R. KowALCZYK , and B. Jedrzejewska . 1997. “Predation of Eurasian Lynx on Roe Deer and Red Deer in Białowieża Primeval Forest, Poland.” Acta Theriologica 42, no. 2: 203–224.

[ece371381-bib-0071] Ordiz, A. , C. Milleret , J. Kindberg , et al. 2015. “Wolves, People, and Brown Bears Influence the Expansion of the Recolonizing Wolf Population in Scandinavia.” Ecosphere 6, no. 12: 1–14. 10.1890/ES15-00243.1.

[ece371381-bib-0072] Pace, M. L. , J. J. Cole , S. R. Carpenter , and J. F. Kitchell . 1999. “Trophic Cascades Revealed in Diverse Ecosystems.” Trends in Ecology & Evolution 14, no. 12: 483–488. 10.1016/S0169-5347(99)01723-1. 10542455

[ece371381-bib-0073] Padié, S. , N. Morellet , A. J. M. Hewison , et al. 2015. “Roe Deer at Risk: Teasing Apart Habitat Selection and Landscape Constraints in Risk Exposure at Multiple Scales.” Oikos 124, no. 11: 1536–1546. 10.1111/oik.02115.

[ece371381-bib-0074] Paradise, C. J. , and W. A. Dunson . 1997. “Insect Species Interactions and Resource Effects in Treeholes: Are Helodid Beetles Bottom‐Up Facilitators of Midge Populations?” Oecologia 109, no. 2: 303–312. 10.1007/s004420050088. 28307184

[ece371381-bib-0075] Pearl, J. 2000. Models, Reasoning and Inference. Vol. 19. Cambridge University Press.

[ece371381-bib-0076] Pop, M. I. , M. A. Dyck , S. Chiriac , et al. 2023. “Predictors of Brown Bear Predation Events on Livestock in the Romanian Carpathians.” Conservation Science and Practice 5, no. 3: e12884.

[ece371381-bib-0077] Pop, M. I. , R. Iosif , I. V. Miu , L. Rozylowicz , and V. D. Popescu . 2018. “Combining Resource Selection Functions and Home‐Range Data to Identify Habitat Conservation Priorities for Brown Bears.” Animal Conservation 21, no. 4: 352–362. 10.1111/acv.12399.

[ece371381-bib-0113] Pop, M. I. , R. Iosif , B. Promberger‐Fürpass , et al. 2025. “Romanian Brown Bear Management Regresses.” Science 387, no. 6741: 1361–1361. 10.1126/science.adv0410 40146822

[ece371381-bib-0078] Popescu, V. D. , R. Iosif , M. I. Pop , S. Chiriac , G. Bouroș , and B. J. Furnas . 2017. “Integrating Sign Surveys and Telemetry Data for Estimating Brown Bear ( *Ursus arctos* ) Density in the Romanian Carpathians.” Ecology and Evolution 7, no. 18: 7134–7144. 10.1002/ece3.3177. 28944005 PMC5606905

[ece371381-bib-0079] Rigg, R. , and M. Gorman . 2004. “Spring‐Autumn Diet of Wolves (*Canis Lupus*) in Slovakia and a Review of Wolf Prey Selection.” Oecologia Montana 13, no. 1–2: 30–41.

[ece371381-bib-0080] Riley, S. J. , S. J. DeGloaria , and R. Elliot . 1999. “Index That Quantifies Topographic Heterogeneity.” Intermountain Journal of Sciences 5, no. 1–4: 23–27.

[ece371381-bib-0081] Ripple, W. J. , and R. L. Beschta . 2006. “Linking a Cougar Decline, Trophic Cascade, and Catastrophic Regime Shift in Zion National Park.” Biological Conservation 133, no. 4: 397–408. 10.1016/j.biocon.2006.07.002.

[ece371381-bib-0082] Ripple, W. J. , and R. L. Beschta . 2012. “Trophic Cascades in Yellowstone: The First 15years After Wolf Reintroduction.” Biological Conservation 145, no. 1: 205–213. 10.1016/j.biocon.2011.11.005.

[ece371381-bib-0083] Ripple, W. J. , J. A. Estes , R. L. Beschta , et al. 2014. “Status and Ecological Effects of the World's Largest Carnivores.” Science 343, no. 6167: 1241484. 24408439 10.1126/science.1241484

[ece371381-bib-0084] Ritchie, E. G. , and C. N. Johnson . 2009. “Predator Interactions, Mesopredator Release and Biodiversity Conservation.” Ecology Letters 12, no. 9: 982–998. 10.1111/j.1461-0248.2009.01347.x. 19614756

[ece371381-bib-0085] Roedenbeck, I. A. , and P. Voser . 2008. “Effects of Roads on Spatial Distribution, Abundance and Mortality of Brown Hare ( *Lepus europaeus* ) in Switzerland.” European Journal of Wildlife Research 54, no. 3: 425–437. 10.1007/s10344-007-0166-3.

[ece371381-bib-0086] Rossa, M. , S. Lovari , and F. Ferretti . 2021. “Spatiotemporal Patterns of Wolf, Mesocarnivores and Prey in a Mediterranean Area.” Behavioral Ecology and Sociobiology 75, no. 2: 32. 10.1007/s00265-020-02956-4.

[ece371381-bib-0087] Salvatori, V. , H. Okarma , O. Ionescu , Y. Dovhanych , S. Find'o , and L. Boitani . 2002. “Hunting Legislation in the Carpathian Mountains: Implications for the Conservation and Management of Large Carnivores.” Wildlife Biology 8, no. 1: 3–10. 10.2981/wlb.2002.002.

[ece371381-bib-0088] Scherber, C. , N. Eisenhauer , W. W. Weisser , et al. 2010. “Bottom‐Up Effects of Plant Diversity on Multitrophic Interactions in a Biodiversity Experiment.” Nature 468, no. 7323: 553–556. 10.1038/nature09492. 20981010

[ece371381-bib-0089] Schweiger, A. H. , I. Boulangeat , T. Conradi , M. Davis , and J. C. Svenning . 2019. “The Importance of Ecological Memory for Trophic Rewilding as an Ecosystem Restoration Approach.” Biological Reviews 94, no. 1: 1–15. 10.1111/brv.12432. 29877019

[ece371381-bib-0090] Shipley, B. 2000. “A New Inferential Test for Path Models Based on Directed Acyclic Graphs.” Structural Equation Modeling 7, no. 2: 206–218.

[ece371381-bib-0091] Shipley, B. 2009. “Confirmatory Path Analysis in a Generalized Multilevel Context.” Ecology 90, no. 2: 363–368. 10.1890/08-1034.1. 19323220

[ece371381-bib-0092] Shurin, J. B. , E. T. Borer , E. W. Seabloom , et al. 2002. “A Cross‐Ecosystem Comparison of the Strength of Trophic Cascades.” Ecology Letters 5, no. 6: 785–791. 10.1046/j.1461-0248.2002.00381.x.

[ece371381-bib-0093] Sin, T. , A. Corrandini , M. I. Pop , et al. 2017. “Wolf (*Canis lupus*) in the Eastern Romanian Carpathians: First Estimates of Population Parameters Based on a Non‐Invasive Integrated Sampling Design, in. 10th Baltic Theriological Conference, Tartu, Estonia.”

[ece371381-bib-0094] Sin, T. , A. Gazzola , S. Chiriac , and G. Rîșnoveanu . 2019. “Wolf Diet and Prey Selection in the South‐Eastern Carpathian Mountains, Romania.” PLoS One 14, no. 11: e0225424. 10.1371/journal.pone.0225424. 31751409 PMC6874069

[ece371381-bib-0095] Sinclair, A. R. 1985. “Does Interspecific Competition or Predation Shape the African Ungulate Community?” Journal of Animal Ecology 54, no. 3: 899–918. 10.2307/4386.

[ece371381-bib-0096] Smith, T. J. , and C. M. McKenna . 2013. “A Comparison of Logistic Regression Pseudo R2 Indices.” Multiple Linear REgression Viewpoints 39, no. 2: 17–26.

[ece371381-bib-0097] Soe, E. , J. Davison , K. Süld , H. Valdmann , L. Laurimaa , and U. Saarma . 2017. “Europe‐Wide Biogeographical Patterns in the Diet of an Ecologically and Epidemiologically Important Mesopredator, the Red Fox *Vulpes Vulpes*: A Quantitative Review.” Mammal Review 47, no. 3: 198–211. 10.1111/mam.12092.

[ece371381-bib-0098] Soliño, M. , B. A. Farizo , and P. Campos . 2017. “Hunters' Preferences and Willingness to Pay for Driven Hunts in Southern Europe.” Wildlife Research 43, no. 8: 649–654.

[ece371381-bib-0099] Stenegren, M. , C. Berg , C. C. Padilla , et al. 2017. “Piecewise Structural Equation Model (SEM) Disentangles the Environmental Conditions Favoring Diatom Diazotroph Associations (DDAs) in the Western Tropical North Atlantic (WTNA).” Frontiers in Microbiology 8: 810. https://www.frontiersin.org/articles/10.3389/fmicb.2017.00810. 28536565 10.3389/fmicb.2017.00810 PMC5423296

[ece371381-bib-0100] Stoessel, M. , B. Elmhagen , M. Vinka , P. Hellström , and A. Angerbjörn . 2019. “The Fluctuating World of a Tundra Predator Guild: Bottom‐Up Constraints Overrule Top‐Down Species Interactions in Winter.” Ecography 42, no. 3: 488–499. 10.1111/ecog.03984.

[ece371381-bib-0101] Strickland, M. S. , D. Hawlena , A. Reese , M. A. Bradford , and O. J. Schmitz . 2013. “Trophic Cascade Alters Ecosystem Carbon Exchange.” Proceedings of the National Academy of Sciences 110, no. 27: 11035–11038. 10.1073/pnas.1305191110. PMC3703983 23776213

[ece371381-bib-0102] Svenning, J.‐C. , P. B. M. Pedersen , C. J. Donlan , et al. 2016. “Science for a Wilder Anthropocene: Synthesis and Future Directions for Trophic Rewilding Research.” Proceedings of the National Academy of Sciences 113, no. 4: 898–906. 10.1073/pnas.1502556112. PMC4743824 26504218

[ece371381-bib-0103] Swenson, J. 2000. “Action Plan for Conservation of the Brown Bear in Europe (*Ursus arctos*).”

[ece371381-bib-0104] Tănăsescu, M. 2019. “Restorative Ecological Practice: The Case of the European Bison in the Southern Carpathians, Romania.” Geoforum 105: 99–108. 10.1016/j.geoforum.2019.05.013.

[ece371381-bib-0105] Terborgh, J. , and J. A. Estes . 2013. Trophic Cascades: Predators, Prey, and the Changing Dynamics of Nature. Island Press.

[ece371381-bib-0106] Terborgh, J. , J. A. Estes , P. Paquet , et al. 1999. “The Role of Top Carnivores in Regulating Terrestiral Ecosystems.” In Continental Conservation: Scientific Foundations of Regional Reserve Networks, 39–64. Island Press.

[ece371381-bib-0107] Trouwborst, A. 2010. “Managing the Carnivore Comeback: International and EU Species Protection Law and the Return of Lynx, Wolf and Bear to Western Europe.” Journal of Environmental Law 22, no. 3: 347–372. 10.1093/jel/eqq013.

[ece371381-bib-0108] Valdmann, H. , Z. Andersone‐Lilley , O. Koppa , J. Ozolins , and G. Bagrade . 2005. “Winter Diets of wolfCanis Lupus and Lynx *lynx lynx* in Estonia and Latvia.” Acta Theriologica 50, no. 4: 521–527. 10.1007/BF03192645.

[ece371381-bib-0109] Valente, A. M. , P. Acevedo , A. M. Figueiredo , C. Fonseca , and R. T. Torres . 2020. “Overabundant Wild Ungulate Populations in Europe: Management With Consideration of Socio‐Ecological Consequences.” Mammal Review 50, no. 4: 353–366. 10.1111/mam.12202.

[ece371381-bib-0110] Wikenros, C. , O. Liberg , H. Sand , and H. Andrén . 2010. “Competition Between Recolonizing Wolves and Resident Lynx in Sweden.” Canadian Journal of Zoology 88, no. 3: 271–279. 10.1139/Z09-143.

[ece371381-bib-0111] Wolf, C. , and W. J. Ripple . 2017. “Range Contractions of the World's Large Carnivores.” Royal Society Open Science 4, no. 7: 170052. 10.1098/rsos.170052. 28791136 PMC5541531

[ece371381-bib-1004] Zimmermann, F. , C. Breitenmoser‐Würsten , A. Molinari‐Jobin , and U. Breitenmoser . 2013. “Optimizing the Size of the Area Surveyed for Monitoring a Eurasian lynx (*Lynx lynx*) Population in the Swiss Alps by Means of Photographic Capture–Recapture.” Integrative Zoology 8, no. 3: 232–243. 24020463 10.1111/1749-4877.12017

[ece371381-bib-0112] Zweifel‐Schielly, B. , M. Kreuzer , K. C. Ewald , and W. Suter . 2009. “Habitat Selection by an Alpine Ungulate: The Significance of Forage Characteristics Varies With Scale and Season.” Ecography 32, no. 1: 103–113.

